# An Optical-Fiber-Based Airborne Particle Sensor

**DOI:** 10.3390/s17092110

**Published:** 2017-09-14

**Authors:** Yifan Wang, John F. Muth

**Affiliations:** Department of Electrical and Computer Engineering, North Carolina State University, Raleigh, NC 27695, USA; ywang52@ncsu.edu

**Keywords:** particle sensor, fiber optic sensor, aerosol detection, scattering measurements

## Abstract

A new optical-fiber-based airborne particle counter is reported. Unlike traditional light-scatter-based techniques, the particle is detected through the drop in optical fiber coupling efficiency as the particle disrupts the electromagnetic mode of the optical beam. The system is simple, substantially smaller than traditional systems, and does not require high power laser input. This makes it attractive for wearable air quality monitors where size is a premium. There is close agreement between theoretical model and experimental results for solid and liquid particles in the 1 to 10 µm range.

## 1. Introduction

Airborne particulate matter (PM) is a complex mixture of extremely small particles and liquid droplets that is usually generated by different forms of combustion or chemical processes, or through mechanical wear [1,2,3]. The sizes of particulates range widely from submicron to ~100 μm, with >10 μm particles settling rapidly on the scale of minutes in still air, while smaller particles remain suspended for longer periods of time. Particulate exposure can be harmful to human health [1]. Several health problems, such as aggravated asthma, irregular heartbeats, and lung cancer have been linked to exposure to particulate matters [4]. Particulates can also act as abrasives or contaminates, and are detrimental to the cleanness and performance of machinery. Generally, regulatory agencies have divided airborne particulates into 4 classifications. PM10—particles less than 10 μm in diameter, PM2.5—particles less than 2.5 μm in diameter, PM1.0—particles less than 1 μm in diameter, and PM0.1—100 nm or less in diameter, and known as ultra-fine particles or nanoparticles. Particles with sizes between PM10 and PM2.5 are sometimes referred to as the coarse fraction and PM2.5 particles and smaller are called the fine fraction.

Traditionally, an optical particle counter (OPC) relies on a focused light beam and ambient/forward particle scattering [5,6]. Using a long optical path, the scattered light can be separated from the main optical beam, detected and counted. Since the energy of the scattered light is very small compared to the original light field, this kind of OPC usually needs a high power laser input, a set of optics to focus the light field, and sensitive light detecting equipment to capture the signal. This makes traditional OPCs relatively large and expensive. Recently, researchers have been interested in lower-cost and compact devices for atmospheric measurements and personal particle monitoring.

Our goal was to investigate methods where long optical path lengths and high laser powers are not required. This would reduce the size and cost of the detection system. In 2013, R.S. Gao et al. reported a high-sensitivity optical particle counter using a single drilled lens [7]. The particle sensing was based upon detecting the forward laser scattering signal instead of perpendicular scattering signal, which resulted in a higher signal amplitude. The lowest detectable size limit could be as small as 100 nm, as suggested by theoretical calculation. In Zhu and Ozdemir’s work [8], particles are driven towards the tapered region of a single mode fiber, as particles adhere to the surface of the fiber, the cladding mode is altered, resulting in signal drops at the signal receiving end. By measuring the number and magnitude of these drops, the particles’ information can be revealed, but the accumulation of particles on the fiber limits long-term use as a sensor. Another example involving optical fiber is the effort towards chip scale sensing by L. Cui, T. Zhang and H. Morgan [9], in which they took advantage of multimode fibers and microfluidic channels to detect both 10 μm and 45 μm bioparticles in a fluid.

In this paper, we describe an optical fiber particle counter where the air flow with particles is guided through the coupling region between two single mode fibers. We avoided using particle scattering as a method, since the desire was to minimize the volume of the sensor package and the amount of energy consumed by the light source. The resulting sensor measures the drop in intensity as the particle disrupts the electromagnetic mode of the light beam. The implementation described here is best suited for measuring the coarse fraction since it has the best performance for particles in the 1 to 10 μm size range. However, since there is increased interest in the measurement of submicron and nano-sized particles, we point out that, with the use of a condensation cell, where smaller particles act as nuclei to form larger-sized liquid particles, the device may also be used to count smaller particles. This condensation technique has been used in commercial particle counting systems to increase the detection of submicron particles.

## 2. Materials and Methods

**Figure 1:**
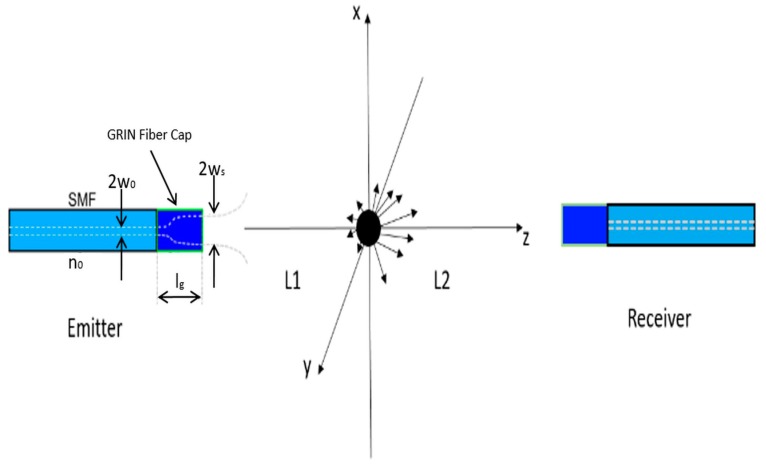
The light from the emitting fiber is a single mode beam, and the receiving fiber is also single mode. When the particle passes through the beam the resulting signal loss in mode coupling is larger than just the geometric occlusion of the size of the particle blocking the beam.

The principle behind this method is simple. However, rather than simple geometric occlusion of light passing through the beam, the passing particles disrupt the electromagnetic mode, changing the coupling between the two fibers. This disruption causes a drop in transmission that can be used to count particles. As shown in Figure 1, two optical fiber probes are placed head to head, with some distance in the middle. The distance between particle and the emitter fiber probe is L_1_ and the distance between particle and the receiver fiber probe is L_2_.

**Figure 2:**
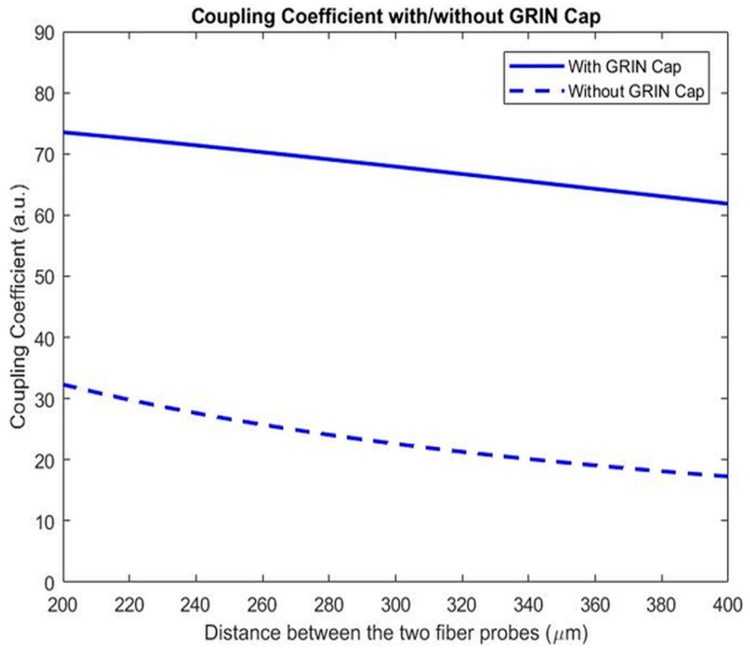
Coupling coefficient with and without GRIN caps spliced to the ends of single mode fibers. The single mode fiber and GRIN fiber’s parameters are from Newport F-SF single mode fiber and Thorlabs GIF-625 graded index fiber.

Each fiber probe is made by fusion splicing a quarter pitch graded index (GRIN) fiber cap onto the end of a single mode fiber. The GRIN caps serve as collimating optics for the output beam from the single mode fibers. The output from a single mode fiber can be roughly seen as a Gaussian beam, without collimating optics, the width of the Gaussian wavefront would enlarge very quickly after leaving the fiber. As a result, only a small fraction of energy from the emitting fiber would reach the receiving fiber, which limits the overall sensitivity of the system. A comparison between the two fiber probes’ coupling coefficients with and without the quarter pitch GRIN caps is as shown in Figure 2. The introduction of GRIN caps greatly increases the coupling coefficients, making it possible to maintain a relatively high coupling efficiency while having a comparatively large separation between the fiber probes, which is important for the particles to efficiently pass through the coupling region and interact with the light beam. Improving the coupling efficiency between the separated fiber probes by fusing a graded index fiber of appropriate length to act as a lens, as shown in Figure 2, has been well established [10,11]. The fiber probe was made by first splicing a single mode fiber and a GRIN fiber together, and then cutting the GRIN fiber to a desired length under a magnifier. In making the fiber, it is important to ensure that there is no air bubble at the splicing junction, and that the GRIN cap’s cross-section after the cut is flat and clean.

At the entrance window of the receiver, according to scattering theory, the total electric field *E_t_* equals the summation of the incident field without perturbation *E_i_* and scattering field *Es* [12]. The incident light field from the emitter fiber can be regarded approximately as a transverse Gaussian wave. Assume the incident light is x-polarized, then *E_i_* = *E_x_*, and

(1) $$\{\begin{array}{l} E_{x}=\frac{w_{s}}{w}E_{0}\exp[-\frac{x^{2}+y^{2}}{w^{2}}]\exp[-i(k(z-z_{0})-\phi_{z}-\phi_{xy})]\\ w_{s}=\frac{\lambda /\sqrt{A}}{\pi w_{0}n_{0}\sqrt{\sin^{2}(\sqrt{A}l_{g})+\frac{\lambda }{\pi w_{0}^{2}n_{0}\sqrt{A}}\cos^{2}(\sqrt{A}l_{g})}} \end{array}$$

where *E*_0_ is the amplitude at the center of the beam waist, *E_x_* and *E_y_* are the electric field amplitudes along *x* and *y* axes, respectively. *w* is the local beam width as the beam propagates. *k* is the wavenumber. *z*_0_ is the position of the emitter probe along *z* axis. *w_s_* is the beam width at the emitting window [13,14,15]. *λ* is the wavelength of the light. 2*w*_0_ and *n*_0_ are mode field diameter (MFD) and core refractive index of the single mode fiber. *A* is called the GRIN constant of the GRIN lens. *lg* is the length of the GRIN cap. *ϕ_z_* and *ϕ_xy_* are functions that describe longitudinal and transverse profiles of the beam. On the other hand, the scattering field could be obtained by applying the generalized Lorenz-Mie theory (GLMT) and far-field approximation, in which

(2) $$\{\begin{array}{c} E_{s\theta }=\frac{E_{0}e^{-ikr}}{-ikr}\cos(\varphi )\sum_{n=1}^{\infty }\frac{2n+1}{n(n+1)}g_{n}(a_{n}\tau_{n}+b_{n}\pi_{n})\\ E_{s\varphi }=\frac{E_{0}e^{-ikr}}{ikr}\sin(\varphi )\sum_{n=1}^{\infty }\frac{2n+1}{n(n+1)}g_{n}(a_{n}\pi_{n}+b_{n}\tau_{n}) \end{array}$$

where *E_sθ_* and *E_sφ_* are electric fields in azimuthal and polar directions, respectively. *r* is the radial distance from the scattering center. The shape factor *g_n_* describes the form of the incident field [16]. *a_n_* and *b_n_* are the Mie coefficients for external scattering amplitude calculation using the below expressions:

(3) $$\{\begin{array}{c} a_{n}=\frac{m^{2}j_{n}(mx)[xj_{n}(x)]\prime -\mu_{1}j_{n}(x)[mxj_{n}(mx)]\prime }{m^{2}j_{n}(mx)[xh_{n}^{\left(1\right)}(x)]\prime -\mu_{1}h_{n}^{\left(1\right)}(x)[mxj_{n}(mx)]\prime }\\ b_{n}=\frac{\mu_{1}j_{n}(mx)[xj_{n}(x)]\prime -j_{n}(x)[mxj_{n}(mx)]\prime }{\mu_{1}j_{n}(mx)[xh_{n}^{\left(1\right)}(x)]\prime -h_{n}^{\left(1\right)}(x)[mxj_{n}(mx)]\prime } \end{array}$$

where *m* is the relative refractive index of the sphere, *x* is the size parameter, *μ*_1_ is the relative magnetic permeability of the sphere, *j_n_* is spherical Bessel function of order n, and $h_{n}^{\left(1\right)}$ is first order Hankel function. The primes denote derivatives with respect to the argument. Transfer expressions in Equation (2) from spherical coordinates to Cartesian coordinates, and sum the incident and scatter fields up, the electric fields after scattering could be expressed as

(4) $$\{\begin{array}{l} E_{x}=E_{i}+E_{s\theta }\cos(\theta )\cos(\varphi )+E_{s\varphi }\sin(\varphi )\\ E_{y}=E_{s\theta }\cos(\theta )\sin(\varphi )+E_{s\varphi }\cos(\varphi ) \end{array}$$

The perturbation in the wavefront, together with energy extinction caused by the presence of the particle, result in a mode mismatch between the emitter and the receiver fiber probes. This mismatch causes coupling efficiency loss that is detected at the other end of the receiver fiber. This loss is larger than the geometric occlusion of the particle in the beam. The normalized energy drop is accounted for by taking the integration of the fiber mode and the distorted wavefront.

(5) $$T=[\frac{2}{\pi w_{0}^{2}}\int_{-\infty }^{\infty }\int_{-\infty }^{\infty }E_{x}(x,y,x){|}_{z\prime =0}\times E_{x\prime }^{*}(x\prime ,y\prime ,z\prime ){|}_{z\prime =0}dx\prime dy\prime {]}^{2}$$

where *T* is the power transmission rate between the two fiber probes with particle scattering. *o* and *o*’ are the origins of two Cartesian coordinate systems centered at the emitting GRIN lens surface and the receiving GRIN lens surface, respectively. *E_x_*’ is the fundamental fiber mode which is extrapolated to the end of the receiving GRIN lens.

**Figure 3:**
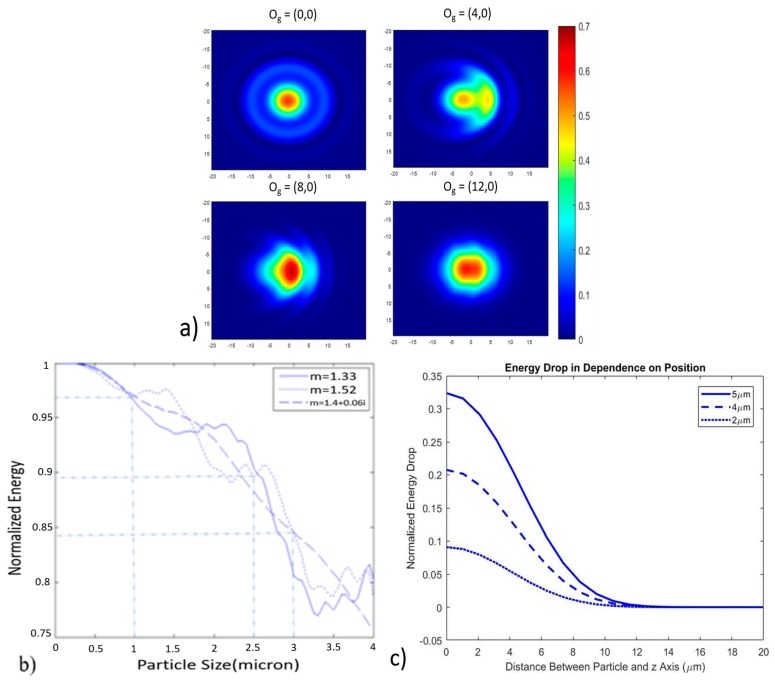
(**a**) Some simulated total forward intensity distribution on the *xy* plane (the wave propagation is along the *z* axis) after scattering by on-axis and off-axis particles (here, on-axis means the particle is on the *z* axis, off-axis means the particle is not on the *z* axis). OG specifies the position of the particle, the unit of the coordinates is μm, the particle being simulated has 2 μm radius and a real refractive index of m = 1.52, laser beam width at beam waist is 14.2 μm, (**b**) calculated coupling energy drop for particles with different refractive indices, the distance between the two fiber probes is 200 μm, laser beam width at waist is 14.2 μm, (**c**) theoretical coupling energy drop for on-axis and off-axis particles with 5 μm, 4 μm and 2 μm diameters, the refractive index m = 1.52.

Figure 3a shows simulated wavefront at the receiving window with both on and off axis particle scattering. Assuming the particle is at the center of the laser’s Gaussian beam profile yields the relationship between particle size and coupling energy drop as shown in Figure 3b. As can be seen in Figure 3b, with >2.5 μm particles, the coupling energy drop is larger than 10%, which could be easily detected by a common photodiode. Figure 3c depicts the relationship between energy drop and distance between particle and *z* axis.

## 3. Results and Discussion

**Figure 4:**
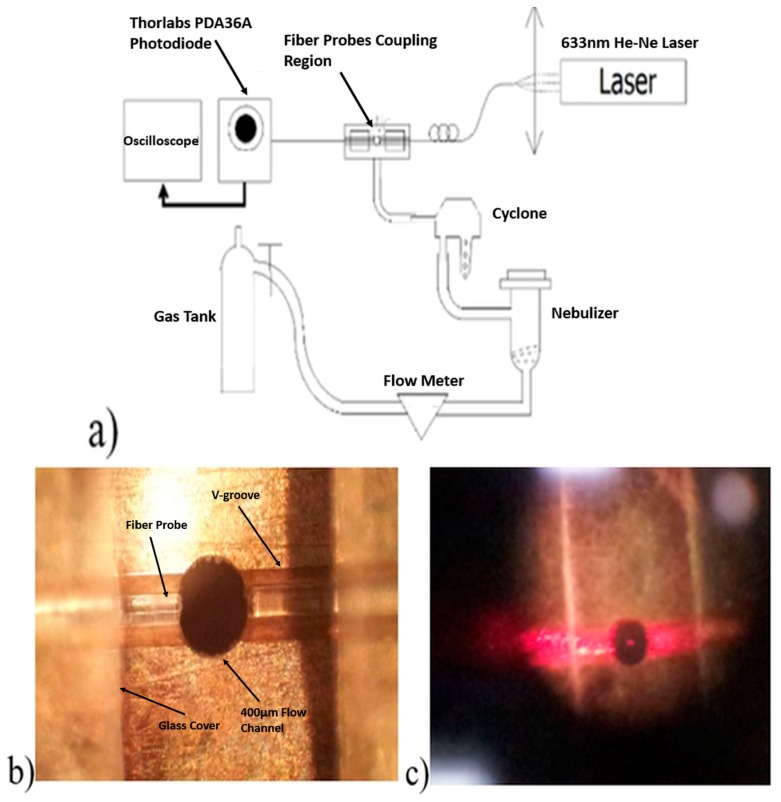
(**a**) Experimental setup allowing generation and size segregation of particles, (**b**) fiber probes in the V-groove holder with 400 μm separation, (**c**) picture taken during experiment, where a low flow rate and large particles are used to enable the scattered light from the particles to be seen.

The transmitting and receiving fiber probes were made by fusion splicing Newport F-SF single mode fibers with Thorlabs graded index GIF-625 fibers using a Sumitomo Type 36 fiber splicer. The graded index fiber was then trimmed to ¼ pitch (246.5 μm length) to act as a focusing and collection lens [17]. The light source used was a 4 mW Newport 633 nm He-Ne laser. The two fiber probes were placed head-to-head in a V-groove carved on the surface of a brass plate, covered by glass plates. Between the two fiber probes, there is a 400 μm diameter hole that allows air flow to pass through. Thus, the distance between the emitter and the receiver probes is also 400 μm. During the experiments, a Newport optical fiber scrambler was used to ensure that there was only one fundamental mode in the emitting fiber probe. A picture of fiber probes in the V-groove is as shown in Figure 4b.

**Figure 5:**
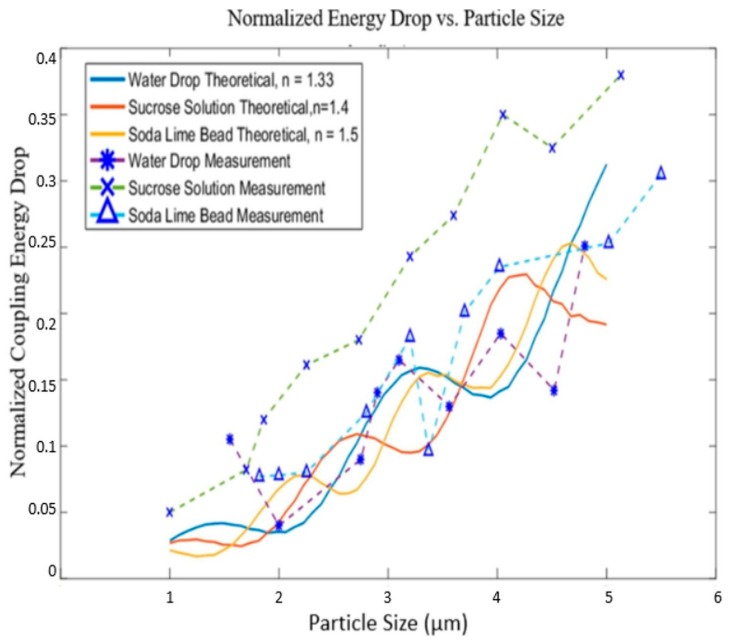
Solid lines are calculations of the loss as a function of particle given refractive index and particle size. The dotted lines connect experimental data where the particle size is determined by the cut-off point of the cyclone at a given flow rate and the largest signal excursions are assumed to be caused by particles with sizes equal to the cut-off plus 0.5 μm.

Three kinds of particles with different refractive indices: water droplets, sucrose solution droplets and soda lime glass beads, were tested. As depicted in Figure 4a, a Mabis CompMist compressor nebulizer kit was used to produce droplets from water and sucrose solutions. Soda lime glass beads were introduced into the air flow through a sealed glass jar which was connected to the system (not shown in the graph). The particle sizes were controlled using an SKC plastic cyclone and a regulator. With a certain flow rate, particles larger than the cut-off size of the cyclone would be filtered out of the air flow. When the air flow rate increases, the cyclone’s cut-off size decreases. Thus, by controlling the flow rate, the largest particle size remaining in the air flow can be determined. In Figure 5, to be conservative, we assume that the largest particle left in the air flow after passing through the cyclone is 0.5 μm larger than the cyclone’s 50% cut-off size.

The signal from the receiving probe was detected by a Thorlabs PDA36A photodiode. A digitizing oscilloscope was connected to the photodiode to show the waveform captured by it. Since the air flow contains a variety of particles with sizes below the cut-off size, for each flow rate, the largest excursion was caused by particles of the maximum size being near the center between the fibers (position 0,0). The percentage of coupling energy drop for a certain sized particle was determined by dividing the amplitude of the largest downward spike by the average amplitude of the signal on the oscilloscope’s output waveform.

Thus, by controlling the system’s flow rate, we could easily measure the percentage of coupling energy drops caused by particles with different sizes. For higher flow rates where smaller particles disrupt the coupling, the drops in transmission are closer to the baseline noise level. For the smallest particles counted the quantization of the digitizing oscilloscope was limiting.

Experiments were performed with water droplets, sugar solution droplets and soda lime glass beads. It was found, as shown in Figure 5, that for water particles and soda lime glass particles—where the index and sizes are known—there was good agreement with the theoretical model. The results of the sucrose solution were about 10% higher than expected, which may be the result of a higher concentration of sucrose in the droplets than expected, or possibly water evaporation during transport. However, it was clear that the sucrose solution particles followed the same trend, and could also be measured. The percentage energy drop is not strictly monotonic along the increase in particle size, which means that within a certain interval, 2 particles with different sizes might cause an identical energy drop. If the desire were to precisely determine the particle size, rather than count the particles, this would require more investigation.

**Figure 6:**
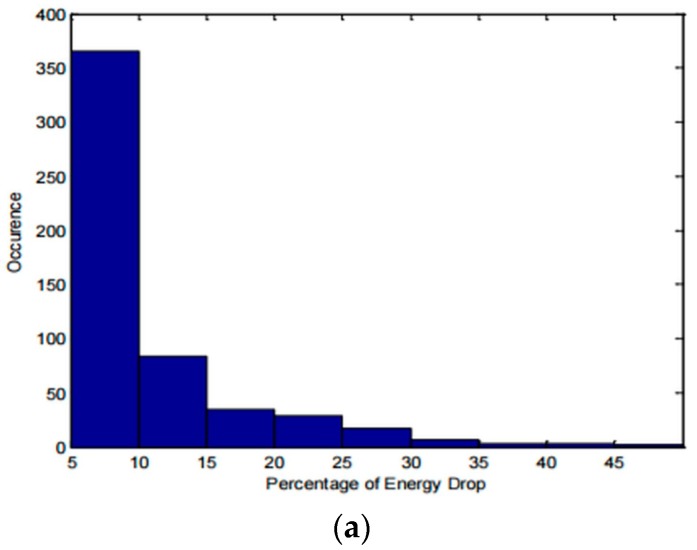

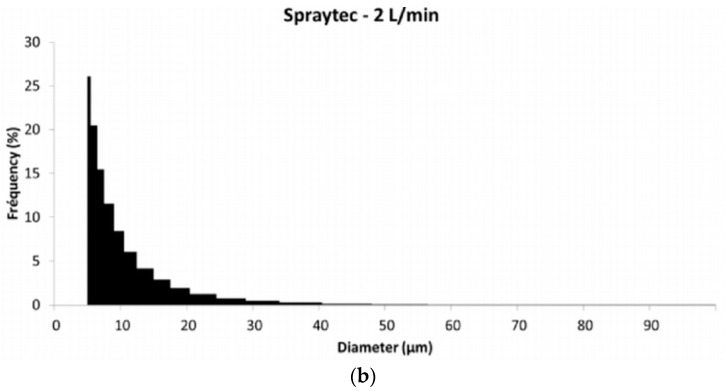
(**a**) Count of coupling energy drops during the test of water particles with our device. Coupling energy drops smaller than 5% are considered noise and excluded from the count; (**b**) Particle size distribution in a nebulizer’s output measured with Malvern’s Spraytec particle counter [18].

In addition to revealing the largest particle size in an air flow, the system may also be used to determine the size distribution of the particles in the air flow. Figure 6a shows the count of percentage energy drops with the sensing volume directly connected to the output of the nebulizer, without cyclone in the system to restrain the size of particles. The particles are water droplets.

Figure 6b is the particle size distribution in a nebulizer’s output measured with Malvern’s Spraytec particle size measurement device. The particles are water droplets, the measurements were taken with a 2 lpm air flow rate. Particles with <5 μm diameters are excluded from this statistical result.

It can be seen that the general trend in Figure 6a agrees with that of Figure 6b [18]. However, the distribution in Figure 6a is skewed towards small coupling energy drops. This is because some of the smaller signals are generated by relatively large particles passing through the periphery of the laser beam; thus, this graph cannot be seen as a direct representation of size distribution in the particle flow. To better understand and extract particle size information from this method, additional studies with calibrated particles need to be performed, and it is likely that the optimization of the air flow to more precisely direct particles could also help. However, at this early stage in development, a size separation method such as cyclone appears to be practical.

In general, large energy drops tend to be more credible than small energy drops. As a result, for a particle mix with certain sized particles, enlarging the distance between the two optical fiber probes would cause a decrease in the corresponding signal strength, which in turn would cause a decrease in sensing accuracy. On the other hand, reducing the distance between the two fiber probes helps to increase the sensing accuracy. However, the distance between the two fiber probes cannot be too small, because of air flow considerations and the increasing likelihood of particle deposition. We found that a distance between the two probes of at least 200 μm was sufficient. Misalignment of the fiber probes can reduce the sensing accuracy of the system, but was minimized by using a v-groove platform, and can be monitored by the average energy reaching the detector. Multiple simultaneous particles could result in a potential counting error, but in flow conditions, we did not observe this to be an issue even with relatively high density of particles. In general, the particles passing though the active volume were separated in time enough that distinct drops in transmission were observed, and coincident detection of particles did not appear to be a problem. For low-cost systems, such as those used for personal or portable particle monitoring, determining the particle size to high precision may be less important than counting the particles within a wider size range where the upper size is determined by a filter at the inlet.

Although demonstrated with particles in the 1–10 μm range, we believe that the system has the potential for sub-micron particle sensing after several simple alterations. These experiments were performed with a He-Ne laser, which has a relatively long wavelength of 633 nm. Switching to a light source with shorter wavelength increases the sensitivity. The curves in Figure 3b were calculated with a wavelength of 633 nm, for a particle with a size of 2.5 μm, the coupling loss was about 10 percent, depending on the particle type. If a 532 nm laser was used, the coupling loss caused by the particle passing would increase to about 20 percent, making the particle easier to count. For shorter wavelengths, a smaller fiber core or specialty fiber may be desired, and coupling the incident laser light into the fiber would be more challenging. We also believe that the micro-optics could be improved by adjusting the length of the GRIN fiber lens, such that the beam waist is located in the middle of the coupling region. Decreasing the distance between the two fiber probes may also improve the sensitivity to mode mismatch, as mentioned above. These changes all help to magnify the signal strength when particles pass through the sensing region, making it easier to identify smaller particles.

The optical sensing portion of this sensor consists of only two single optical fibers and a brass plate to hold them in position, and is of very small size (~0.3 mm^3^) compared to the long optical path lengths used in many traditional particle sensing devices. Diode lasers pigtailed to single-mode optical fibers are also relatively small and compact, as are PIN photodiodes. We estimate that, with some optimization, the optical system and electronics excluding the air handling could be approximately 3 cm × 3 cm × 1.5 cm. Thus, this configuration has the potential to be integrated into multipurpose sensing systems as the particle sensing section, as well as being developed into a wearable individual particle counter.

## 4. Conclusions

This was a tabletop demonstration of a compact particle sensor that allowed flexibility in testing and observation of particles going through the system. This setup showed good agreement between theoretical calculation and experimental results. The optical portion of the system is very, very small, with a volume of approximately 450 μm × 450 μm × 150 μm, or about 0.3 mm^3^. While optical fibers were used in this case to provide a well-defined optical mode, other approaches could also be used. The small sensing volume compared to traditional air particle monitors (which use scattering, and require relatively long optical path lengths, as well as relatively high-power light sources) makes the approach attractive for applications where small size is highly valuable. This approach can be extended to shorter wavelength light sources to further increase the sensitivity. Similarly, depending on the air and particle handling geometries, one can also expect higher resolution if particles are more precisely guided, as is done in commercial systems. The small size also potentially allows this approach to be used in conjunction with other particle detection methods.
